# Brown Recluse Spider Bite Superimposed With Methicillin-Resistant Staphylococcus aureus Infection: A Case Report

**DOI:** 10.7759/cureus.90379

**Published:** 2025-08-18

**Authors:** John Stauffer, Jalal Ibrahim, Shivang Patel, Matthew Thomas, Urvish Patel

**Affiliations:** 1 College of Medicine, Lake Erie College of Osteopathic Medicine, Bradenton, USA; 2 Dermatology, Lake Erie College of Osteopathic Medicine, Bradenton, USA; 3 Pediatrics and Child Health, Lake Erie College of Osteopathic Medicine, Bradenton, USA; 4 Internal Medicine, Ascension Health, Jacksonville, USA

**Keywords:** brown recluse spider bite, dermonecrosis, envenomation, loxoscelism, mrsa infection

## Abstract

Brown recluse spider (BRS) bites are known for their potential to cause localized dermonecrosis and, in severe cases, systemic complications such as loxoscelism. However, secondary infections following envenomation are rare and can dramatically alter the clinical course. We present the case of a 52-year-old male who developed progressive cellulitis, tissue necrosis, and abscess formation after a confirmed BRS bite to the left leg. Imaging revealed extensive subcutaneous fluid collections, and surgical drainage confirmed superimposed methicillin-resistant *Staphylococcus aureus* (MRSA) infection. The atypical features of this case, including timing of presentation outside the typical seasonal pattern and the development of a secondary bacterial infection, highlight the need for clinicians to recognize and manage unusual complications of BRS bites. Prompt diagnosis, aggressive antibiotic therapy, multidisciplinary consultation, and surgical intervention were critical in preventing further morbidity. This case emphasizes the importance of considering secondary infections in patients with worsening symptoms following envenomation.

## Introduction

Brown recluse spider (BRS), also known as the *Loxosceles reclusa* spider, is very common in the south, west, and midwest areas of the United States, where they can mostly be located in attics, basements, or other dark areas where humans would not expect and thus get bitten [1]. A bite from the BRS can have a wide range of consequences, from asymptomatic to severe sepsis and possible amputation/death. Some outcomes of getting bitten by them are called dermonecrotic arachnidism, which is essentially local tissue injury caused by the spider's venom itself, and also loxoscelism, which is the progression nature of the venom and bite, leading to severe septic shock and systemic consequences [1]. Other outcomes of a BRS bite are septic shock, disseminated intravascular coagulation (DIC), renal failure, hematological abnormalities, and necrotizing fasciitis [2]. Our patient proved to be a unique case with a BRS bite, as later on, they also had a superimposed methicillin-resistant *Staphylococcus aureus* (MRSA) infection alongside the previous bite. Cases such as these are more likely to lead to worse outcomes and require more attention, like our patient did. This case was worked up in Florida, where, although BRS bite cases are found in the south, not many were reported in the Florida region itself. We present a case of a 52-year-old male who presented to the emergency department (ED) due to a spider bite on his left posterior leg, which led to a MRSA infection, causing an unusual presentation.

## Case presentation

A 52-year-old male with a past medical history of hypertension presented to the ED with complaints of redness, swelling, and pain in the left posterior knee and thigh. Symptoms began approximately five days prior when he felt what he believed to be an insect bite, followed by progressive erythema, swelling, and worsening pain. Initial evaluation included an ultrasound, which revealed an ill-defined fluid collection. His white blood cell count was 11.8 k/µL. He was treated with antibiotics and discharged home.

**Figure 1:**
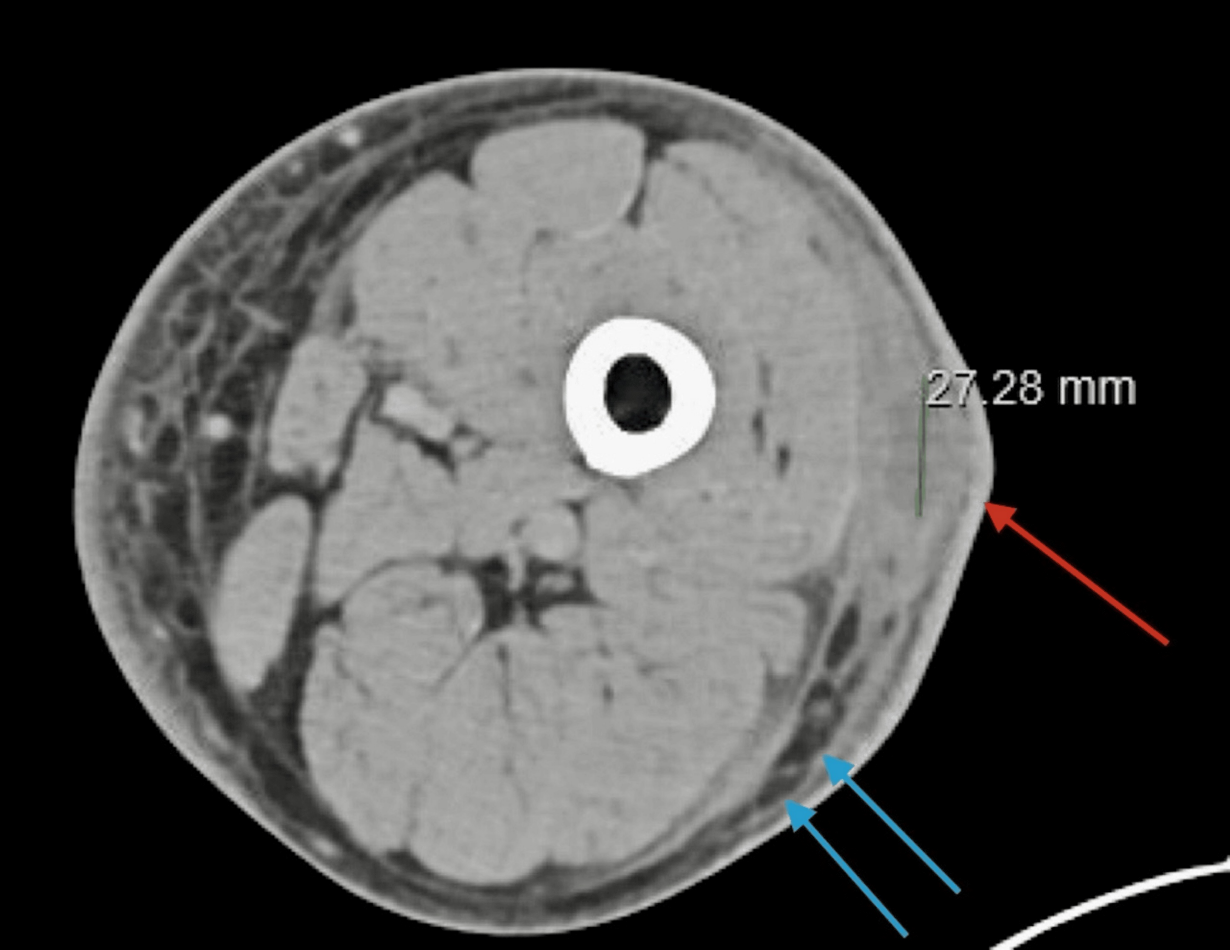
CT impression: Diffuse subcutaneous edema in the thigh. There is a superficial fluid collection in the mid-lateral thigh, approximately 17 cm cranial to the joint space measuring up to 2.7 x 5.5 x 2.1 cm (red arrow). There is also fluid tracking inferiorly toward the level of the knee joint in the posterior lateral subcutaneous tissues measuring up to 3.9 x 12.0 x 2.3 cm (blue arrow). Findings likely reflect abscess in the setting of clinical concern for cellulitis.

The patient returned to the ED shortly after due to increasing pain. A computed tomography (CT) scan demonstrated moderate subcutaneous cellulitis with a questionable fluid collection (Figure 1). The patient was admitted, and he was started on intravenous vancomycin, and both Infectious Disease (ID) and Orthopedics were consulted. Despite antibiotic therapy, his cellulitis progressed with worsening pain and swelling. Clindamycin and sulfamethoxazole-trimethoprim were added to broaden coverage. He was subsequently diagnosed with sepsis, mild transaminitis, and anemia.

The patient later confirmed that he remembered being bitten by a BRS on the left leg. The patient’s wife also found the spider shortly after admission, expressing physical characteristics of a BRS, such as the dark brown, violin-shaped marking on the cephalothorax. The affected area showed progression of cellulitis with increasing blistering and necrosis. Magnetic resonance imaging (MRI) of the left leg was ordered, and General Surgery and Wound Care were consulted. Wound care evaluation noted superficial tissue necrosis at sites of prior blistering, with the skin appearing red and firm, extending proximally toward the hip. Scattered small blisters and drainage were also noted.

**Figure 2:**
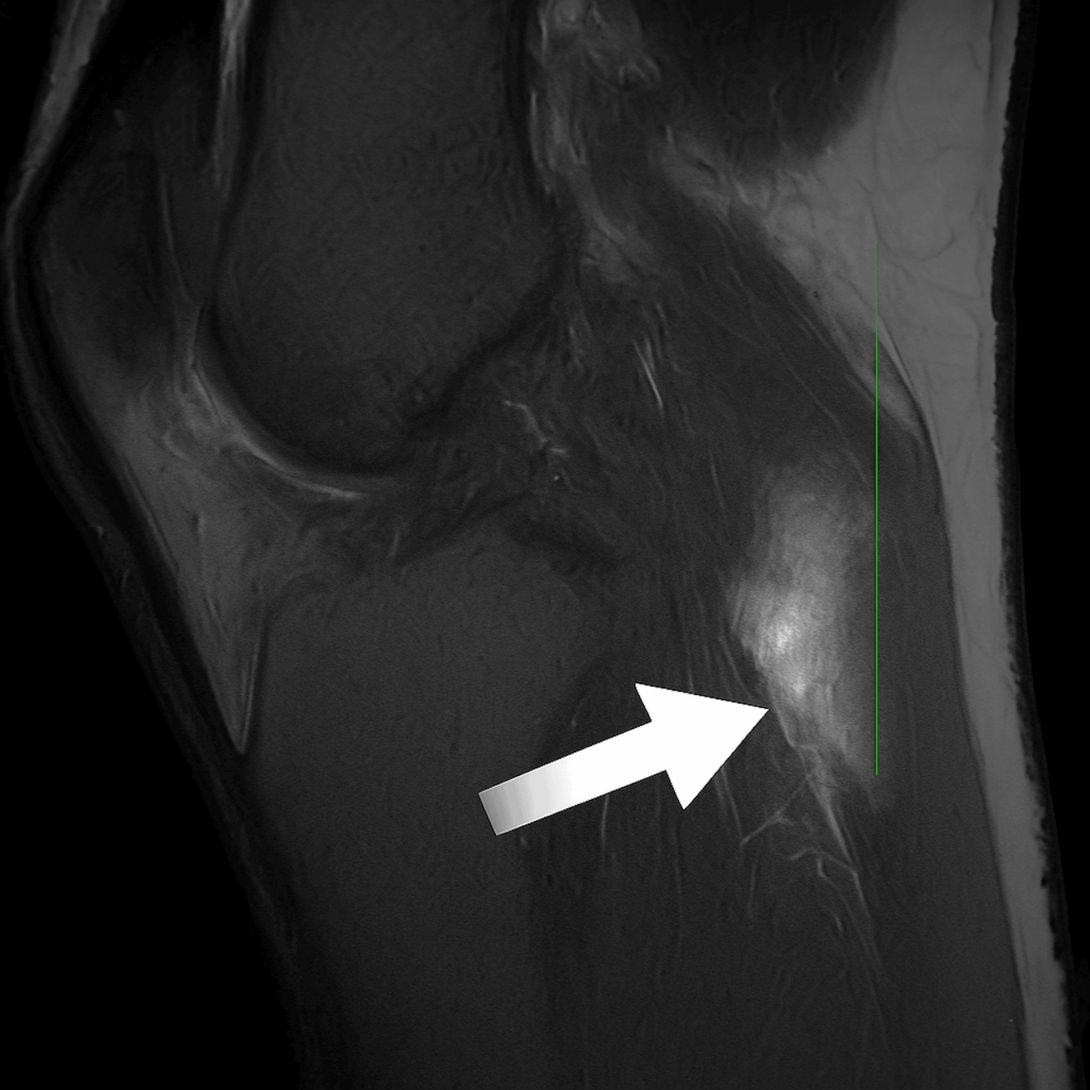
MRI results showed a 5.2 x 1.3 x 6.6 cm soft tissue fluid collection just deep to the skin along the posterolateral left knee (indicated with the arrow). MRI, magnetic resonance imaging.

Due to concern for necrotizing fasciitis, surgical evaluation was performed. MRI revealed a 5.2 x 1.3 x 6.6 cm soft tissue fluid collection just beneath the skin in the posterolateral knee region, along with regional soft tissue edema (Figure 2). No abnormal bone signal was observed. Antibiotics were escalated to linezolid and sulfamethoxazole-trimethoprim, with noted improvement in edema and erythema.

The patient underwent incision and drainage (I&D) of the abscess. Fluid culture was taken, and after three days, it grew MRSA, unknown whether it was acquired in the hospital or from the community, and a drain was placed. Orthopedics determined that a left knee aspiration was not indicated. On discharge, the patient was prescribed oral linezolid.

## Discussion

BRS cases are typically seasonal, ranging from summer to early winter, and are a helpful diagnostic tool for physicians to use when prompted with a potential bite [3]. Interestingly, the patient got the BRS bite mid-April, going out of the typical range of the usual cases, giving this a unique and variable presentation of how these complications occur. Diagnosing a BRS is usually based on clinical presentation. The diagnosis of a spider bite can be confirmed if the patient or someone close to the patient (spouse or family member) was able to see the spider bite themselves and an entomologist was able to catch and identify the spider, or if there is a clinical picture of a spider bite [1]. Laboratory workup is unnecessary except in cases of systemic symptoms, especially in children. Spider bites from a presumed arachnid can be confused for a BRS bite [1]. Several clinical features can help differentiate other causes of skin lesions from a true BRS bite. BRS typically presents as a single, flat lesion with a pale center due to localized ischemia and minimal swelling, unless on the face or feet [1]. They rarely exceed 10 cm, do not ulcerate immediately (usually 7-14 days later), and are not exudative [1]. In contrast, lesions that are numerous, significantly swollen, elevated over 1 cm, ulcerate early, or persist beyond three months are unlikely to be caused by a BRS [1].

BRS bites can present in four distinct clinical forms. The first is a mild, unremarkable reaction with minimal local tissue damage that heals on its own [2]. The second involves a small lesion accompanied by surrounding redness and itching [2]. The third and more serious form includes localized skin necrosis [2]. The fourth and most severe presentation involves systemic effects, such as vascular complications and DIC [2]. The clinical presentations of BRSs are mainly triggered by sphingomyelinase D, a toxic component of their venom, which can activate the hosts' leukocytes [4]. Sphingomyelinase D induces a systemic inflammatory reaction similar to that observed in an endotoxic shock from bacteria [4]. The sphingomyelinase D toxin depletes serum hemolytic complement, prolongs activated partial thromboplastin time, and reduces levels of clotting factors VIII, IX, XI, and XII [5]. This leads to rapid coagulation and blockage of small capillaries, ultimately resulting in tissue necrosis [5]. Our patient presented with up to the third clinical form of RBS. This patient's presentation is consistent with a BRS bite due to its solitary lesion, slow onset of ulceration with progressive necrosis, minimal initial swelling, and lack of exudate. 

Although the patient did develop localized progression of cellulitis with increasing blistering and necrosis of the left posterior knee and thigh, the patient's MRI demonstrated subcutaneous fluid collection with regional soft tissue edema, which is not typical for a BRS bite. While brown recluse venom causes localized dermal and subcutaneous necrosis, it usually results in dry necrosis, ulceration, and inflammation, not significant fluid collections or abscess formation [1]. What makes this case atypical for a brown recluse bite is the large fluid collection (5.2 x 1.3 x 6.6 cm) on imaging, which correlates with a secondary bacterial infection. In this case, the fluid cultures grew MRSA as the secondary infection. 

This patient had a BRS bite that was superimposed with MRSA, giving a unique and rare presentation of the typical BRS bites that are usually reported. It is imperative for clinical providers to not only properly diagnose a BRS bite, but also to be open to any potential superinfections a patient may have, such as MRSA, to give appropriate medications and treatment. 

## Conclusions

This case highlights a rare and clinically significant complication of a BRS with a superimposed MRSA infection, resulting in progressive cellulitis, necrosis, and abscess formation requiring surgical intervention. While BRS envenomation typically presents with localized necrosis and dry ulceration, the presence of a large fluid collection and positive MRSA culture suggests a secondary bacterial infection that altered the typical clinical course. This underscores the importance of maintaining a broad differential when evaluating presumed spider bites, especially in cases with systemic symptoms or atypical findings on imaging. Early recognition, appropriate antimicrobial coverage, and timely surgical management are crucial in preventing severe outcomes in such complex presentations.
